# Mechanical and Material Properties of Mortar Reinforced with Glass Fiber: An Experimental Study

**DOI:** 10.3390/ma14030698

**Published:** 2021-02-02

**Authors:** Marcin Małek, Mateusz Jackowski, Waldemar Łasica, Marta Kadela, Marcin Wachowski

**Affiliations:** 1 Faculty of Civil Engineering and Geodesy, Military University of Technology, ul. Gen. Sylwestra Kaliskiego 2, 01-476 Warsaw, Poland; marcin.malek@wat.edu.pl (M.M.); mateusz.jackowski@wat.edu.pl (M.J.); waldemar.lasica@wat.edu.pl (W.Ł.); 2 Building Research Institute (ITB), ul. Filtrowa 1, 00-611 Warsaw, Poland; 3 Faculty of Mechanical Engineering, Military University of Technology, ul. Gen. Sylwestra Kaliskiego 2, 00-908 Warsaw, Poland; marcin.wachowski@wat.edu.pl

**Keywords:** glass waste, glass fiber, recycling, eco-efficient mixture, fiber-reinforced mortar, mechanical properties, compressive strength, flexural strength, split tensile strength

## Abstract

The progressive increase in the amount of glass waste produced each year in the world made it necessary to start the search for new recycling methods. This work summarizes the experimental results of the study on mortar samples containing dispersed reinforcement in the form of glass fibers, fully made from melted glass waste (bottles). Mortar mixes were prepared according to a new, laboratory-calculated recipe containing glass fibers, granite as aggregate, polycarboxylate-based deflocculant and Portland cement (52.5 MPa). This experimental work involved three different contents (600, 1200, and 1800 g/m^3^) of recycled glass fibers. After 28 days, the mechanical properties such as compressive, flexural, and split tensile strength were characterized. Furthermore, the modulus of elasticity and Poisson coefficient were determined. The initial and final setting times, porosity, and pH of the blends were measured. Images of optical microscopy (OM) were taken. The addition of glass fibers improves the properties of mortar. The highest values of mechanical properties were obtained for concrete with the addition of 1800 g/m^3^ of glass fibers (31.5% increase in compressive strength, 29.9% increase in flexural strength, and 97.6% increase in split tensile strength compared to base sample).

## 1. Introduction

The amount of recycled glass is small compared to its annual production. Due to the high usage of glass for packaging purposes, the recycling method currently available on the market is not efficient enough to fully process all the waste generated. Some of the packaging is also not suitable for traditional recycling. Therefore, there is a need to search for alternative recycling methods [1,2,3]. About 50% of all the packaging in the world is single-use [4,5], hence it generates various types of waste that takes hundreds of years to decompose. This leads to serious environmental problems that could result in health issues, animal life degradation, or even water pollution [6,7,8]. Materials that cannot be reused in the food industry alternatively could be processed, in order to later be incorporated into concrete. This would make the material that accounts for 3% of the world’s total energy consumption more environmentally friendly [9].

Nowadays, concrete additives are being derived from recycling or special treatment of waste materials [10,11,12]. Waste should be recycled at first, but their usage in the concrete mix recipe is definitely possible [13,14,15]. For example, ash, old tires, foil or plastic packaging, glass bottles [16,17,18] can get a second life as a component of a concrete facade, foundation, or beam of a building. The waste materials in the form of fibers, e.g., steel, glass, polypropylene [19,20,21], available on the market and obtained using mechanical and thermal treatment can work very well as reinforcement in concrete [22,23,24], ultimately preventing cracks from progressing [25,26]. Fiber-type additives are widely used in various types of composites, not only concrete ones (e.g., polymer), and they are showing promising research results [27,28,29].

The rapidly growing market of polymer composites is making good use of glass fibers. Currently, over 95% of reinforced composite products have glass fiber due to the low cost of use. This in turn has created a high demand, which has contributed to the evolution of glass fiber production in recent years, simultaneously now allowing the production of fibers in any other shape or size. The most important parameter determining the production and its success is the size of the fibers [30,31,32].

During the production of fibers, including those from recycling, a thin surface coating, the sizing, mainly made of polymeric materials is applied [33,34,35]. This type of coating is applied to almost all types of man-made fibers during their manufacture. Sizings are indispensable auxiliaries in the fiber production process. They influence the properties of the fiber-polymer interphase. Only the achievement of an optimized composite interphase in the final composite ensures that the required mechanical parameters of the final composite are met [30,31].

Glass fiber reinforced composite materials were used in several industrial applications due to excellent mechanical properties, no corrosion, as well as lifetime maintenance costs [36]. Khan et al. [37] showed positive glass fiber influence on parameters of composite. The mechanical and thermal properties of epoxy laminates with different fibers (carbon and glass) were tested. Both tensile strength and modulus as well as strain% increased with the increase in fiber content. The tensile strength and modulus of epoxy laminates modified with carbon fibers were remarkably higher than laminates modified with glass fibers, regardless of their compositions (around four times higher). In comparison to the base samples, an average three times increase in these parameters was noted for samples modified with glass fibers (60% modification). Nhut et al. [38] investigated a new glass fiber reinforced polymer for use in bridge structures and buildings. The stiffness of the connections of new polymer increased by about 90% after the reinforcement with eight layers of glass fiber. A significant improvement in the final load on the connections for all three types of modifications was determined. The final loads increased as the number of glass fiber layers grew. After toughening, the average end loads increased approximately 1.7, 2.2, and 2.6 times for the two, four, and eight layers of glass fibers respectively.

Despite the great interest in polymer composites usage in construction, concrete is still the main building material. For this material, there has been a significant amount of research from involvement of modifications to the use of glass fiber as well [39,40,41]. Compared to non-modified concrete, glass fiber modified concrete showed better results in mechanical performance and properties as reported by Sanjeev and Sai Nitesh [42]. The increase in compressive, split tensile, and flexural strength in combination with decrease in slump fall were noticed. The highest increase (20% increase in compressive strength, 13.9% increase in split tensile strength, and 17.7% increase in flexural strength) was reported for the highest quantity of glass fibers used (0.04% addition). Higher values of mechanical properties of concrete modified with glass fiber also were obtained by Moghadam and Izadifard [43]. They reported 19.2% increase in tensile strength and 2.6% increase in compressive strength for 6250 g/m^3^ of glass fiber usage as an addition to concrete mix. About 3% increase in compressive strength of samples with 1.0% by weight of glass fibers (mixed length 6–12–18 mm, diameter 14 μm, and tensile strength >1.7 GPa) was achieved by Ali et al. [44]. For different type of glass fibers, Atewi [45], after 28 days, obtained compressive strength equal to around 74.2 MPa, using glass fibers with length 12 mm, diameter 13 μm, and tensile strength 3.4 GPa, which is around 3% increase as well (compared to non-modified samples with compressive strength equal 71.8 MPa).

Opposite results were reported by Venkata Krishna Bhargava et al. [46]. The 4% decrease in compressive strength, 14% decrease in split tensile strength, and 13% in flexural strength for concrete modified with glass fibers was obtained. However, addition of fly ash was used in the concrete mixture, which may lower the properties of hardened concrete due to high water absorption and take away the water necessary for the cement hydration process.

This study’s goal is to determine the effect of glass fiber addition on the compressive, flexural and split tensile strength of reinforced mortar. One type of recycled glass fiber was tested. The fibers were made from glass fully made of recycled glass bottles. Therefore, presented research results may allow to develop a new glass waste disposal method.

## 2. Materials

### 2.1. Specimen Preparation

All designed mixtures were based on Portland cement, which was CEM I 52.5R, in accordance with EN 197-1:2012 [47], and tap water. Chemical composition of cement and its physical and strength properties are presented in Table 1, which were determined according to EN 196-6:2019-01 [48] and PN EN 196-1:2016-07 [49]. Additionally, polycarboxylate deflocculant as a hardening admixture was used [50].

**Figure 1:**
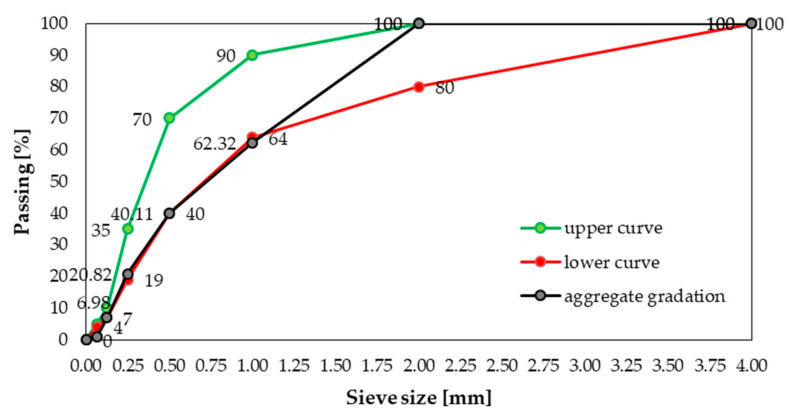
Gradation of crushed granite aggregate.

As a filler, crushed granite aggregate with fraction up to 2.0 mm was used (Figure 1). The filler is heterogeneous (grain size index *C_U_* = 6.23 and *C_C_* = 0.95) and well compacted [52]. Upper and lower curves were determined in accordance with the EN 12620 + A1:2010 [53] standard for natural aggregate with fraction up to 4.0 mm.

Increase in the workability of the mix with significantly reduced amount of water used was accomplished by the addition of chemical admixtures to fiber-reinforced mortar. Maintaining the water-cement ratio w/c at 0.44 was possible, by adding a low-alkaline liquid chemical admixture, free of chlorine, and based on an aqueous solution of modified polycarboxylic ethers (melamine and silanes/siloxanes). In addition, the polymer setting of the cement paste was delayed and the phenomenon of “bleeding” limited. Table 2 presents the chemical composition of the admixture.

### 2.2. Glass Fibers

**Figure 2:**
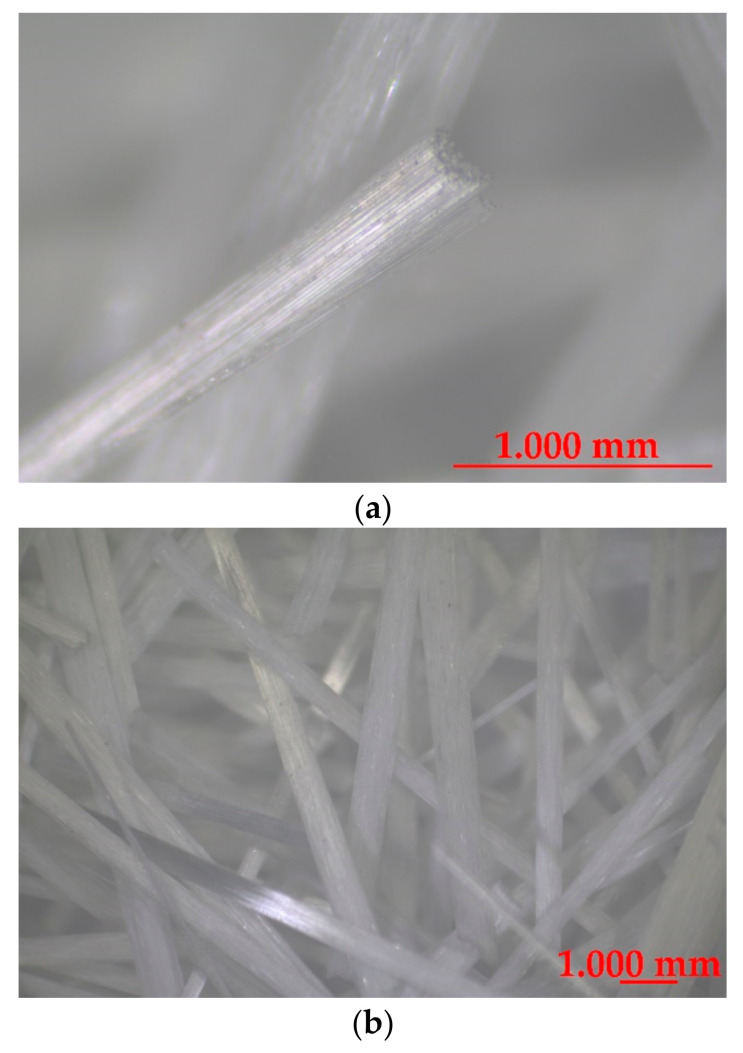
Glass recycled fibers: (**a**) one fiber, (**b**) a few fibers.

Glass fibers from waste materials were used (Figure 2). Fibers were made from the same type of product-glass packaging (bottles) and they are chemically identical.

Glass fibers used in this study were of about 49 mm length (49.4 ± 0.5 mm) were used. The density of fibers is about 2.7 kg/m^3^. The tensile strength and elastic modulus of fibers is 1.7 and 72 GPa, respectively. The basic properties of the tested fibers are presented in Table 3.

### 2.3. Mix Composition

Four different types of mortar mixtures (one without fibers-base mix (BM)—and the rest with different content of fibers) were produced (Table 4). The glass fiber content was 600, 1200, and 1800 g/m^3^ respectively, marked in the article as 600GF, 1200GF, and 1800GF, respectively. For all mixes, constant w/c = 0.44 ratio was used.

### 2.4. Mix Production

After mixing all of the other components for 2 min, the fibers were added to the mortar mix. The whole mixing process lasted five minutes. After that, the samples were formed in molds with dimensions depending on the test, and then compacted on a vibrating table. All samples were produced in laboratory conditions (21 °C temperature and 50% humidity) and stored in water according to EN 12390-2:2019-07 [54].

## 3. Methodology

### 3.1. Test on Concrete Mix

The slump cone test was carried out according to EN 12350-2:2019-07 [55]. Furthermore, the initial and final setting times were measured with the Vicat apparatus (Merazet, Poznań, Poland). Five samples for each mixture were tested.

Air content and pH value of mix were determined according to EN 12350-7:2019-08 [56] and PN-B-01810:1986 [57], respectively. Five samples for each mixture were investigated.

The indicated tests were performed immediately after the process of mixing in the order in which they were listed. The presented values for each tests are the average values of five samples for each mix.

### 3.2. Test on Hardened Concrete Samples

The density of samples of hardened mortar, sized 150 × 150 × 150 mm was determined according to EN 12390-7:2019-08 [58]. Further, the mechanical properties of the hardened mortar samples (compressive strength and flexural strength) were determined using a Zwick machine with force range 0-5000 kN (Zwick, Ulm, Germany). Compressive strength was measured according to EN 12390-3:2019-07 [59] on samples size 100 × 100 × 100 mm. Flexural strength was tested in a three-point bending set-up, on samples size 40 × 40 × 160 mm (beams) in according to EN 12390-5:2019-08 [60]. The nominal distance between the supports was set to 100 mm, allowing horizontal movement by the rollers. Finally, split tensile strength was tested on cylinder shaped samples (0.15 m diameter and 0.30 m height) according to EN 12390-6:2011 [61].

Modulus of elasticity and Poisson’s ratio were determined on cylindrical samples with a diameter of 150 mm and a height of 300 mm according to EN 12390-13:2014-02 [62]. Two 100 mm long resistance strain gauges were bonded halfway up, on two opposite sides of the samples. In order to evaluate the modulus of elasticity, the stress-strain characteristic (*σ-ε* characteristic) was recorded, see Equation (1). Prior to the test, the surfaces directly exposed to compressive stress were ground to ensure the parallelism of the surfaces. The longitudinal and transverse linear displacements were measured with Epsilon extensometers (Epsilon, Jackson, WY, USA). The samples were loaded three times and the load was removed in the lower and upper stress range (according to the characteristic compressive strength). The lengths of the measuring bases of the devices and the recorded values of linear displacements made it possible to determine the deformations for the lower and upper stress range in the tested mortar samples:

(1) $$E=\frac{\Delta \sigma }{\Delta \varepsilon }.$$

**Figure 3:**
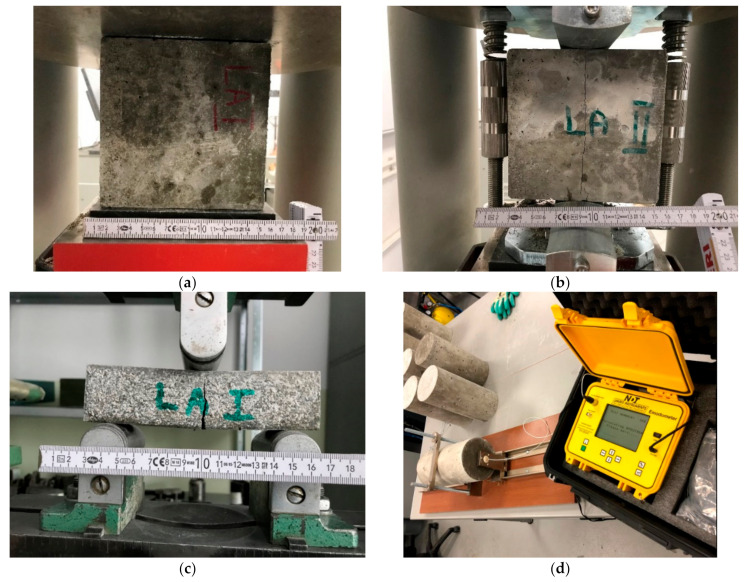
Photographic documentation of tests carried out: (**a**) compressive strength test, (**b**) split tensile strength test, (**c**) flexural strength test, (**d**) elastic modulus test.

Each test of the hardened mortar samples was performed on ten samples of each mixture and the values presented are average values (Figure 3).

The above tests were all carried out after 28 days of curing process. The presented values are the average values of ten samples for each mix.

## 4. Results and Discussion

### 4.1. The Slump Cone Test

The slump cone test results are presented in Table 5. All mortar mixes were classified as S1 according to EN 12350-2:2019-07 [55]. Considering the test results individually, it can be seen that despite the similarities, a slight effect on the consistency can be noticed. Increasing the content of fibers in the mortar mixes resulted in gradual decrease in a slump cone. The same conclusion was made by Sanjeev and Sai Nitesh [42]. They noticed a 23.6% decrease for samples modified with glass fibers (0.04% addition) compared to non-modified samples. Kasagani and Rao [63] also obtained lower results of slump cone tests for all the samples containing glass fibers. They tested different lengths (3.0, 6.0, 12.0, and 20.0 mm) and quantities (0.1, 0.2, 0.3, 0.4, and 0.5% addition) of fibers and in every single case, there was a decrease in slump cone fall. However, the decrease in the slump cone achieved (maximum decrease for 1800GF sample ~31.8% compared to BM) had no effect on the final classification.

### 4.2. The Initial and Final Setting Time

**Figure 4:**
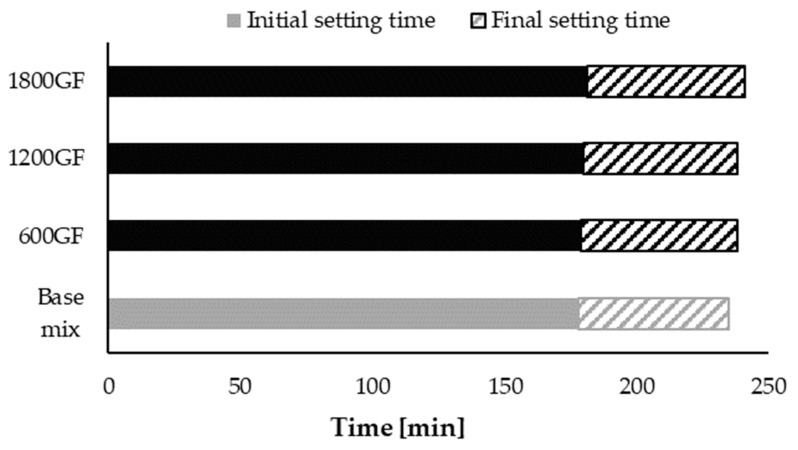
Initial and final setting times distribution of tested mixes: GF marked in black, base mix (no fibers) marked in dark grey.

Initial and final setting time distribution for four mixes (one without fibers and the rest with different content of fibers) are presented in Figure 4. Initial and final setting time distribution for base mix (no fibers) was marked in dark grey, while all modified mixes (containing GF) were marked in black. The highest initial and setting times were obtained for 1800GF, but the occurring difference fits within the error limit of the measurement method. Therefore, the conclusion can be made that addition of glass fiber to the cement mix has no effect on initial and final setting times.

**Figure 5:**
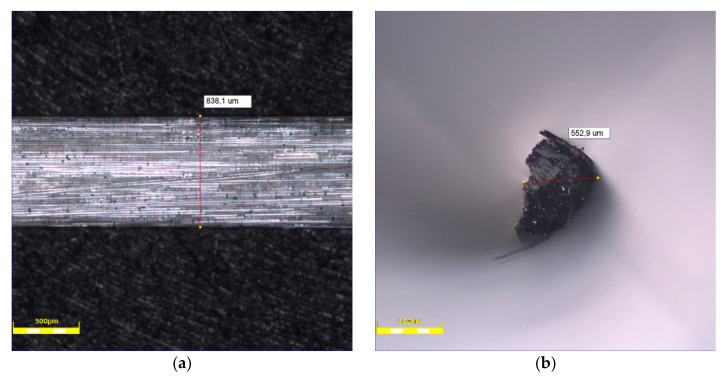
Optical microscope images of glass fiber: (**a**) surface, (**b**) cross-section.

The influence of the fibers’ external structure is not apparent in tests. The surface of GF is smooth and without roughness (Figure 5), therefore no process of forming agglomeration during mixing was observed. The fibers were mixed smoothly and were distributed evenly in the mixture. Neither did they float to the surface nor sink to the bottom. This outcome may be the result of chemical admixture addition.

### 4.3. Air Content

**Figure 6:**
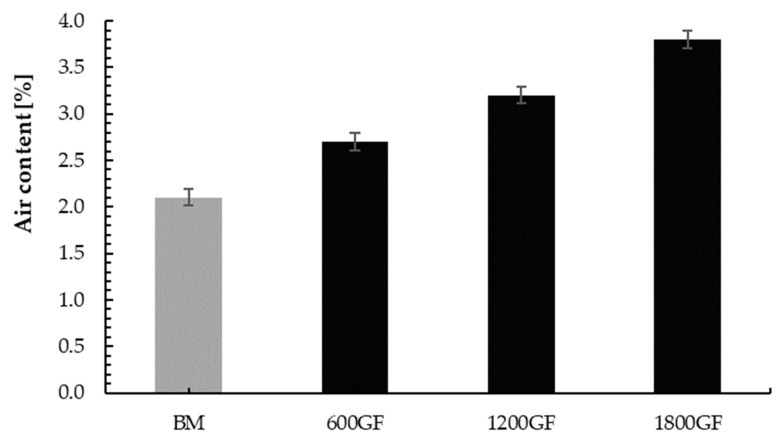
Air content of tested mixes: GF marked in black, base mix (no fibers) marked in dark grey.

The presented test results of the air content (Figure 6) showed that the quantity of glass fibers slightly affects the quantity of pores in the mortar mix. With increase of glass fibers in the mortar, the quantity of pores increases. Similar regularity was shown by Vafaei et al. [64]. He noticed that air content increases slightly with 0.3% addition of polypropylene and polyvinyl alcohol fibers. However, by using 0.5% fiber addition, air content increased by 12% and 20%, respectively, for polypropylene and polyvinyl alcohol fiber and authors stated that this is due to entrapped air voids in fresh mixture as a result of orientation and distribution difficulties of fibers.

In this study, the highest increase was observed for 1800GF samples (80.9% compared to BM).

### 4.4. The pH Test

Based on presented results (Table 6), the pH of all recipes exhibits the alkaline reaction. Highest value of pH was obtained for 1800GF samples (12.97), which is around 1% higher than pH value of BM (12.83). As all presented results are almost similar, glass fiber addition did not affect the pH of the mortar mix significantly. However, during the mortar production and its care, the mortar mix did not reach the melting temperature of the glass fibers or the softening temperature, which can affect pH value. 

### 4.5. Density

The results of the density tests are in the range of 2209 ± 2 kg (for BM) to 2223 ± 2 kg (for 1800GF). After adding 600, 1200, and 1800 g/m^3^ of glass fibers, the density of the mortar increased by 0.57%, 0.60%, and 0.66%, respectively. With the fiber content increase, a density slightly increased. This trend was showed by Madhkhan and Katirai [65]. They reported increase in 5.7 and 18.5 kg/m^3^ in density, which is approximately equal to the obtained results (12–14 kg/m^3^).

### 4.6. Compressive Strength

**Figure 7:**
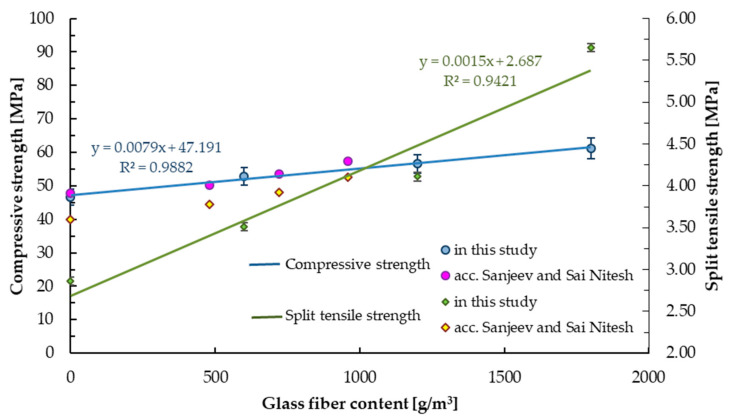
Compressive and split tensile strength.

**Figure 8:**
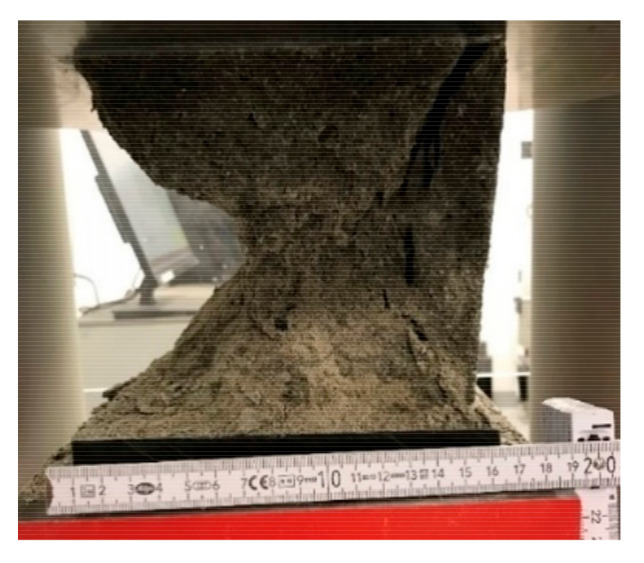
Destruction of sample with 1800 g/m^3^ glass fiber addition in the compressive strength test.

The compressive strength test results for the samples with the 600, 1200, and 1800 g/m^3^ addition of glass fibers are presented in Table 7. The results were compared with the base mix (without fibers). All modified samples showed higher compressive strength values, compared to samples without glass fibers (13.6% increase for 600GF, 21.4% increase for 1200GF, and 31.5% increase for 1800GF compare to BM), see Figure 7. The highest value of compressive strength was obtained for 1800GF samples (61 MPa). The addition of fibers in concrete improved the compressive strength of mortar. The same conclusion was reported by Moghadam and Izadifard [43]. They obtained a 2.6% increase in compressive strength of modified samples (54.77 MPa) compared to non-modified concrete (53.38 MPa), but they used 6250 g/m^3^ glass fibers (which is significantly more than in this study). The fibers are used to bridge micro cracks, thus delaying sample destruction (Figure 8).

### 4.7. Split Tensile Strength

The results of the split tensile strength for samples containing glass fibers in the amount of 0, 600, 1200, and 1800 g/m^3^ are presented in Table 7. The highest values of the split tensile strength were obtained for the sample with the highest glass fibers content (1800 g/m^3^). Linear increase in the split tensile strength can be observed with the increase in glass fiber content (22.7% increase for 600GF, 43.7% for 1200GF, and 97.6% for 1800GF samples), see Figure 7. The same result was also reported by Sanjeev and Sai Nitesh [42]. They obtained maximum increase in split tensile strength (13.9%) occuring with the highest content of glass fibers. However, all tested samples containing glass fibers showed higher split tensile strength results than the base mix samples (3.78, 3.92, and 4.10 MPa for 0.02, 0.03, and 0.04% addition of glass fibers, respectively). Moghadam and Izadifard [43] obtained 19.2% increase in split tensile strength for 6250 g/m^3^ of glass fiber, which is almost 10 times higher than 600GF content of fibers. However, split tensile strength equal to 3.61 MPa was lower by 63.9%. It may be due to the use of glass fibers with different modulus of elasticity.

### 4.8. Flexural Strength

**Figure 9:**
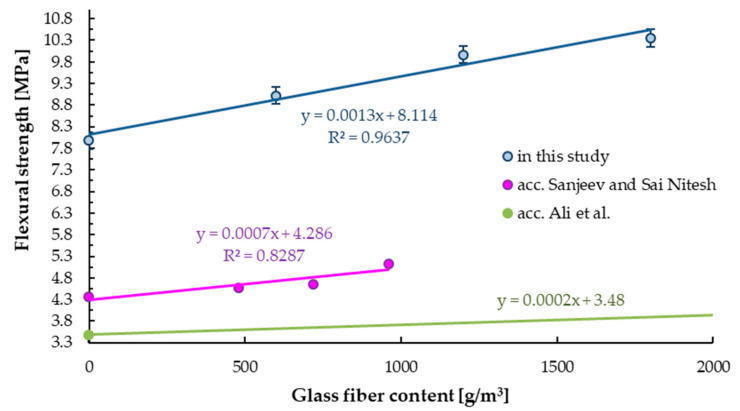
Flexural strength of samples with and without (base mix) glass fibers.

Table 7 shows the results of the flexure strength of tested samples with and without glass fibers, marked black and dark grey, respectively. The highest bending strength values were obtained for samples containing 1800 g/m^3^ of glass fibers (10.4 MPa). Linear increase in flexural strength can be observed with increasing glass fiber content. For samples containing 600, 1200, and 1800 g/m^3^ of glass fibers, an increase by 13.2%, 25.1%, and 29.9% in bending strength was obtained, see Table 7. The same trend was already reported by Ali et al. [66] and Sanjeev and Sai Nitesh [42], see green and pink line in Figure 9. Sanjeev and Sai Nitesh [42] demonstrated that samples with the highest glass fiber content showed the highest flexure strength, with all the modifications having higher results than for base samples. The maximum increase was 17.7% for fibers in the amount of 0.04% (which corresponds to the range between 600–1200 g/m^3^).

### 4.9. Modulus of Elasticity and Poisson Coefficient

The obtained results for modulus of elasticity tests ranged from 31.5 ± 0.4 to 32.1 ± 0.3 GPa (Table 8). It can be observed a slight increase in elastic modulus (1.8% for the 1800GF samples, 1.3% for the 1200GF samples, and 0.8% for the 600GF samples compared to BM). Atewi et al. [45] also reported an increase in the modulus of elasticity for concrete with glass fiber from 0 to 1.5% and with different ratios of nanosilica additions.

The results of the Poisson’s ratio are presented in Table 8. The addition of glass fiber in the range from 600 to 1800 g/m^3^ did not affect the Poisson’s ratio.

## 5. Conclusions

The study shows an improvement in mechanical properties for recycled glass fiber reinforced mortar. Recycled glass fiber from glass packaging (bottles) in the amount of 600, 1200, and 1800 g/m^3^ was used. The following conclusions can be drawn from this study:

- Increasing the fiber content in the mortar mix, the slump cone decreased, but it had no effect on the final classification;
- Air content of reinforced mortar increased linearly with increase of glass fiber content;
- The increase in compressive strength by 13.6% for 600GF, 21.4% for 1200GF, and 31.5% for 1800GF compared to the base sample was determined;
- The improvement in split tensile strength of 97.6%, 43.7%, 22.7% was obtained for samples with 1800, 1200, and 600 g/m^3^ glass fiber compared to the reference sample;
- The flexural strength increased by 13.2%, 25.1%, and 29.9% with an increase in glass fiber by 600, 1200, and 1800 g/m^3^, respectively;
- The use of glass fibers causes a slight increase in mortar elasticity modulus compared to base samples (values higher by 0.8–1.8%);
- The addition of glass fiber in the range from 600 to 1800 g/m^3^ did not affect the Poisson’s ratio.

This work is the result of a wider cycle of research involving the development of environmentally friendly mortar recipes which show increase in their mechanical properties. The set goal of this case study, which was to determine mechanical and material properties of glass fiber reinforced mortar, was successfully achieved. All presented recipes have great application capacity in the current construction industry as a potential new way of glass waste disposal while maintaining or even increasing mortar properties.

## Figures and Tables

**Table 1:** Chemical composition of the cement [51].

Compositions		SiO_2_	Al_2_O_3_	Fe_2_O_3_	CaO	MgO	SO_3_	Na_2_O	K_2_O	Cl
Unit (vol. %)		19.5	4.9	2.9	63.3	1.3	2.8	0.1	0.9	0.05
Specific surface area (m^2^/kg)		400								
Specific gravity (kg/m^3^)		3080–3180								
Compressive strength after days (MPa)	2 days	40–47								
	28 days	67–77								

**Table 2:** Chemical composition of the admixture.

Compositions	O	Na	Si	K
Unit (vol. %)	77.7	14.9	4.8	2.6

**Table 3:** Properties of the glass fibers.

Type of Fiber	Average Thickness [μm]	Average Width [μm]	Length [mm]
Glass fiber (GF)	838.0 ± 10.0	554.8 ± 0.5	48.8–50.3

**Table 4:** Mix proportions (1 m^3^)**.**

Mix Symbol	Cement [kg]	Water [kg]	Aggregate [kg]	Fiber Content [g/m^3^]
BM	468	207	1598	0
600GF				600
1200GF				1200
1800GF				1800

**Table 5:** The slump cone test results.

Mix Symbol	Fiber Content [g/m^3^]	Slump Cone [mm]	Consistency Class [56]
BM	0	22 ± 1	S1
600GF	600	20 ± 2	S1
1200GF	1200	18 ± 1	S1
1800GF	1800	15 ± 2	S1

**Table 6:** The pH test results.

Mix Symbol	Fiber Content [g/m^3^]	pH [–]
BM	0	12.83 ± 0.03
600GF	600	12.86 ± 0.03
1200GF	1200	12.93 ± 0.04
1800GF	1800	12.97 ± 0.03

**Table 7:** Strength parameters of tested samples.

Mix Symbol	Fiber Content [g/m^3^]	Compressive Strength [MPa]	Split Tensile Strength [MPa]	Flexural Strength [MPa]
BM	0	47 ± 1	2.86 ± 0.03	8.0 ± 0.1
600GF	600	53 ± 1	3.51 ± 0.05	9.0 ± 0.1
1200GF	1200	57 ± 1	4.11 ± 0.03	10.0 ± 0.2
1800GF	1800	61 ± 2	5.65 ± 0.04	10.4 ± 0.1

**Table 8:** Modulus of elasticity and Poisson coefficient.

Mix Symbol	Fiber Content [g/m^3^]	Modulus of Elasticity [GPa]	Poisson Coefficient [GPa]
BM	0	31.5 ± 0.4	0.12 ± 0.03
600GF	600	31.8 ± 0.3	0.12 ± 0.04
1200GF	1200	31.9 ± 0.4	0.12 ± 0.03
1800GF	1800	32.1 ± 0.3	0.12 ± 0.04

## Data Availability

Data is contained within the article.
